# A Model Based on Environmental Factors for Diameter Distribution in Black Wattle in Brazil

**DOI:** 10.1371/journal.pone.0100093

**Published:** 2014-06-16

**Authors:** Carlos Roberto Sanquetta, Alexandre Behling, Ana Paula Dalla Corte, Sylvio Péllico Netto, Aurelio Lourenço Rodrigues, Augusto Arlindo Simon

**Affiliations:** 1 Federal University of Parana, Department of Forest Sciences, Curitiba, Paraná, Brazil; 2 TANAC S.A. – Forest Department, Montenegro, Rio Grande do Sul, Brazil; University of Catania, Italy

## Abstract

This article discusses the dynamics of a diameter distribution in stands of black wattle throughout its growth cycle using the Weibull probability density function. Moreover, the parameters of this distribution were related to environmental variables from meteorological data and surface soil horizon with the aim of finding a model for diameter distribution which their coefficients were related to the environmental variables. We found that the diameter distribution of the stand changes only slightly over time and that the estimators of the Weibull function are correlated with various environmental variables, with accumulated rainfall foremost among them. Thus, a model was obtained in which the estimators of the Weibull function are dependent on rainfall. Such a function can have important applications, such as in simulating growth potential in regions where historical growth data is lacking, as well as the behavior of the stand under different environmental conditions. The model can also be used to project growth in diameter, based on the rainfall affecting the forest over a certain time period.

## Introduction

The diameter distribution of a forest permits the characterization of the available wood stock prior to harvest, providing information to support the decision-making process [1]. Moreover, it is a simple and effective means to describe the structure of a stand of trees and the properties that comprise [2]. Clutter et al. [3] point out that the diameter distribution allows us to estimate the number of trees per hectare by diameter class and also to determine the average height in each class. This in turn permits detailed information about the structure of the stands yield potential.

According to Loetsch et al. [2], the diameter distribution indicates the growth stock, which enhances the ability to draw conclusions about the structure of the forest. For black wattle Finger [4] concluded that Johnson’s SB function best described the diameter distribution structure. Meanwhile Maestri [5] used the Weibull distribution to evaluate current and future wood and bark yields, and obtained satisfactory results. Corte et al. [6] found that the Weibull distribution function with 3 parameters best describes the diameter distribution in *Populus* sp.

Machado et al. [7] cite the importance of research on fitting probability density functions in order to represent the per unit area frequency distributions for various forest types, both for native forests and plantations. Thus, the study of the diameter distribution, derived by fitting probability density functions, becomes a relevant aspect to planning forestry activities, in addition to being an effective tool to predict tree growth.

Levins [8] describes three properties which are desirable in a model: precision, accuracy and generality. Normally one of these conditions is not fully met, while a highly acceptable level can be reached for the remaining two. According to Burkhart [9], in the case of traditional models, more emphasis is given to precision, while generality is of less importance.

However, developing more general models that can improve the existing ones is of interest, considering their capacity to predict the economic potential of a plantation as a function of a variety of environmental conditions. In many cases, studies have reported the definition of variables and coefficients numerically expressing the growth of plantations as a function of environmental variables (soil and meteorological elements, among others) [10]–[14]. Meanwhile other authors have taken environmental variables and added them to the same empirical models, assuming that the inclusion of them increases the possibility to make more general models [15]–[17].

In this sense, the present study aimed to study the effects of forest age on the evolution of the diameter distribution in stands of black wattle by a probability density function. Additionally one of the objectives was to correlate the estimators of this function with meteorological elements and soil chemical properties.

## Materials and Methods

To conduct this study we used data from temporary plots installed in commercial plantations of black wattle in regions of high concentrations of plantations in the state of Rio Grande do Sul, in the municipalities of Cristal and Piratini. In each municipality stands were studied in an age sequence: one, three, five and seven years to represent a full cultivation cycle. Table 1 displays the number of trees measured per age and location.

**Table 1:** Number of black wattle trees measured per age and location in Rio Grande do Sul State, Brazil.

Age (years)	Measured trees		
	Cristal	Piratini	Total
1	112	107	219
3	100	106	206
5	90	90	180
7	77	83	160
Total	379	386	765

The plots in the municipality of Cristal are located 30°59′59″ South and 52°02′54″ West and in Piratini they are located 31°26′52″ South and 53°06′14″’ West, at altitudes between 320 and 370 meters above sea level.

For the two regions, plantations were established both in new land (first rotation) and in recovering plantation land (second rotation). For all cases, soil preparation was done along the planting rows (minimum plantation system) and ripped using three plows to 40 cm deep and two harrowing applications. Plantings were spaced at 3×1.75 meters (1,904 plants per hectare) for year one and at 3×1.5 meters for all other ages (2,222 plants per hectare) and 50 grams of NPK (5-30-15) per plant was added immediately after planting.

In each stand a North-facing slope was selected where one plot was demarcated in the each of the upper, middle and bottom thirds of the slope. The size of the plots measured 9×16 meters for the one-year old stands and 9×14 meters for all other ages, making four rows of 10 plants in each row.

The circumference at breast height was measured for every plant in the plots using tape with millimeter graduations. In addition, an evaluation of the chemical characteristics of the surface horizon (0–20 cm) was conducted in the plots. To accomplish this, three samples were collected in each plot, using an auger, which were then combined to yield a single mixed sample for the stand. These final samples were sent to the Soil and Plant Tissue Analysis Laboratory at the *Universidade Regional Integrada do Alto-Uruguai e das Missões* in the city of Frederico Westphalen, RS, to determine the following variables: pH, SMP Index (Derived from the method of Shoemaker, Mac lean e Pratt), clay, organic matter, phosphorus, potassium, aluminum, calcium, magnesium, aluminum + calcium, Cation Exchange Capacity (CEC) at pH 7, CEC base saturation and CEC aluminum saturation.

Also, historical meteorological data series were obtained: maximum, minimum and average air temperature, relative humidity, rainfall and number of hours of solar irradiation representing the ages of the stands. The data were obtained from INMET (National Institute of Meteorology) Pelotas Climatological Station. This was the station closest of the study site during the time the research was being conducted, about 85 km from the municipality of Cristal and 70 km from Piratini.

The global incident radiation was estimated using Angstrom’s equation, modified by Prescot and Penman following the mathematical advancement presented by Vianello and Alves [18] and with coefficients adjusted for the municipality of Pelotas by Steinmetz et al. [19].

### Probability Density Functions (PDF) Tested

The following PDFs were tested in order to describe the diameter frequency: Gamma (two parameters), Log Normal (two parameters), Normal, and Weibull 2p and 3p. In several scientific studies involving diameter distributions of native species has been verified that the behavior of the diameter variable tends to normality, however in some circumstances occur cases of non normality, characterizing conditions of slight asymmetry. Thus, the distributions gamma, log normal and weibull have been those which best detect such asymmetry, and that is why they also have been tested in this case.

All functions were fitted using the procedure described in *Proc capability* in SAS [20], performed for each age of the stand (1, 3, 5 and 7). The software procedure requires the input of raw data and specification of the classes’amplitude, which in this case was two centimeters, opting for fixed intervals (always starting from one centimeter) and number of classes according to the diameter amplitude obtained at each age of the stand. The used fitting method was the maximum likelihood.

Selection of the best probabilistic function was performed after applying the Kolmogorov-Smirnov test at 5% probability. Also an Anderson Darling and Cramer-Von Mises test was also applied to the selected model, ensuring proper use of the probability density function.

### Data Analysis

The effect of age on the shape and evolution of the distribution curves of diameters was evaluated using measures of skewness and kurtosis, as well as the mean, minimum and maximum values.

The asymmetry was evaluated according to the following aspects:

Symmetric: mode = median = arithmetic mean.
Asymmetric to the right or negative if: mode<median<arithmetic mean.
Asymmetric to the left or positive if: mode>median>arithmetic mean.

The asymmetry coefficient is considered moderate if the mode lies between 0.15 and 1, while it is considered strong if greater than 1.

The kurtosis is the degree of flatness or relative height of a distribution, usually with respect to the Normal distribution. Pearson [21] and Pearson [22] defined three types of curves to describe kurtosis:

Leptokurtic: distribution with a relatively high peak and negative excess, i.e., coefficient of kurtosis <0.263.
Platykurtic: curve is more flattened, with positive excess, i.e. coefficient of kurtosis >0.263.
Mesokurtic: intermediate curve, with kurtosis coefficient equal to 0.263.

After choosing the best fitting function, and in order to compare the curves, i.e., whether there is a significant difference between them, we applied the Kolgomorov-Smirnov (KS) statistical test. The test was applied to all combinations of stand age, i.e.: 1 and 3; 1 and 5; 1 and 7; 3 and 5; 3 and 7, and 5 and 7 years, with significance set at 1% probability.

Finally, we performed a simple linear regression analysis, using minimum least square method, between the parameters of the selected function of stand age and meteorological and soil chemical attributes to establish their correlation. To accomplish this, the selected probability function was fit for each planting site, as well as for each age of the planting, in order to obtain the fit estimators for the eight stands.

The relationship between the parameters of the selected function and the mentioned environmental variables was also analyzed by the stepwise variable selection technique. Draper and Smith [23] report that Stepwise Multiple Regression is highly recommended for effectively selecting explanatory variables.

The models adjusted by this method were tested according to the regression requirements, using the White test (homoscedasticity), Shapiro-Wilk test (normality) and Durbin-Watson test (independence). For the multiple models, a tolerance level was determined and hence the variance inflation factor (VIF), since this is an indicator of the effect that other independent variables have on the standard error of a regression coefficient. High VIF values (greater than 10) indicate a high degree of collinearity or multicollinearity [24], or increasing variance in the coefficients given by the correlation between the independent variables. Moreover the significance of the coefficients as well as the coefficients of determination at 1% probability was established by the *t* test.

*Dummy* variables were added to the model chosen to describe the estimators of the probability density function, which allowed us to identify whether relationships between the dependent variables (estimators of the probabilistic function) and the independent variables (variables selected by the *stepwise* method) was the same between the two planting sites. Firstly, the *dummy* variables take on value of either 0 or 1 representing the planting site, i.e.: Di = 1 if the tree were present at site i, and Di = 0, if the tree was absent at site i. This method made it possible to express the individual fitted regressions for both sites to be evaluated as a function of a multiple linear regression, represented by the independent variables in the selected equation such that: EFDP = f (x, Di; Di.x), where EFDP = estimators of the probability density function; x = variables selected by the *stepwise* method, Di = *Dummy* (planting site, where i = 1, municipality of Cristal, i = 2, municipality of Piratini); Di.x = interaction variable Di with the variable x.

To verify the performance of the estimated parameters of the probabilistic function by means of the model obtained via *stepwise*, curves were generated and these were compared with those obtained via direct fitting of the probabilistic function by the *Proc capability* procedure. For this, we used the Kolgomorov-Smirnov test.

## Results and Discussion

**Figure 1:**
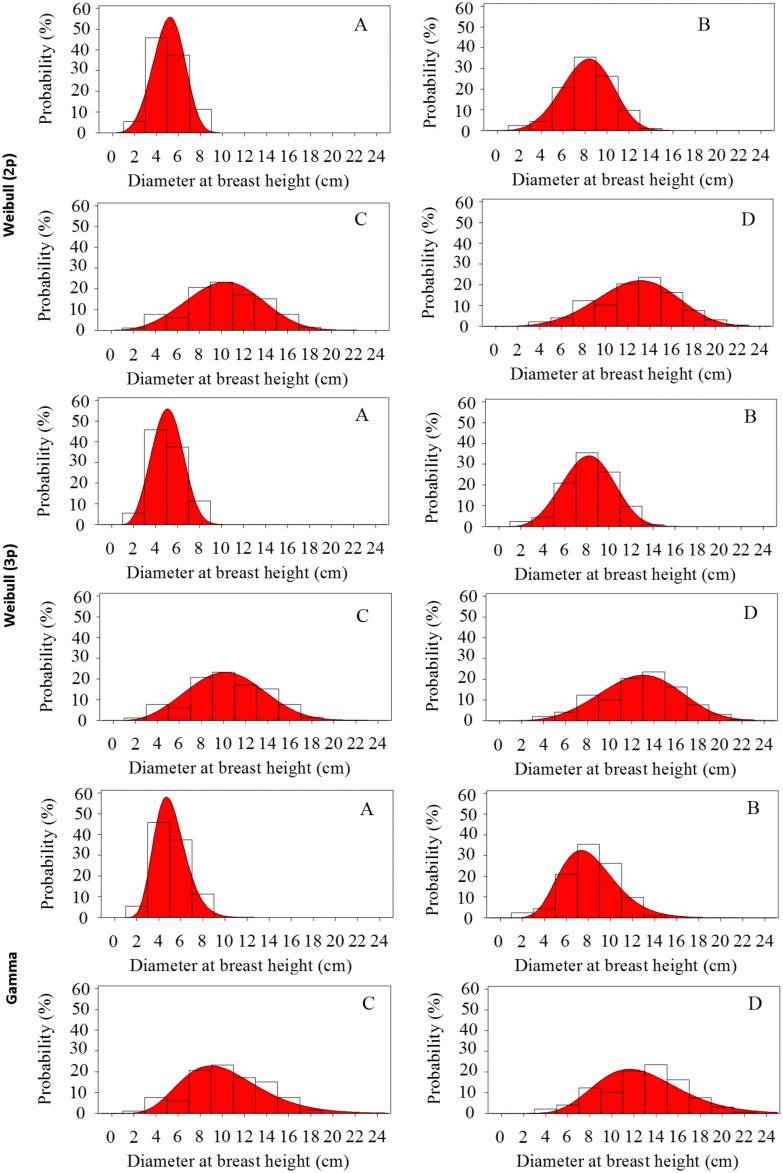
Observed and predicted values for the Gamma function, Weibull 2p and 3p of the probability of occurrence of trees as a function of diameter class in stands of black wattle at one year old (A), three (B) five (C) and seven (D) years old.

The goodness of fit test applied to different PDFs for each age is shown in Table 2. Of all the PDFs tested by the Kolmogorov-Smirnov test, only the Gamma and Weibull functions (2p and 3p) were flexible enough to represent the diameter distributions for all ages (Figure 1), since the values of these tests were not significant. This condition indicates that the observed and expected frequencies are not statistically different from each other, and therefore the null hypothesis could not be rejected. On the other hand, significant values indicate inadequate estimates of diameter frequencies. Due to the sensitivity of this test to any deviation of the central value or dispersion, it has been used to evaluate the goodness of fit produced in the theoretical distribution of a data set [25] and widely used in forestry to determine diameter distributions [1], [6], [26], [27].

**Table 2:** Kolmogorov-Smirnov test of the 19 PDFs to describe the frequency of diameters in one, three, five and seven year old stands of black wattle.

Distribution	Year1	Year3	Year5	Year7
Gamma	0.058^ns^	0.069^ns^	0.047^ns^	0.067^ns^
Log-normal	0.090*	0.109*	0.101*	0.109*
Normal	0.060*	0.070*	0.042^ns^	0.053^ns^
Weibull (2p)	0.050^ns^	0.060^ns^	0.048^ns^	0.048^ns^
Weibull (3p)	0.062^ns^	0.044^ns^	0.042^ns^	0.040^ns^

Where: ns = not significant at the 5% probability level, and *significant at the 5% probability distribution according to Kolmogorov-Smirnov test.

Since the aim of this study was to correlate the estimators of a probability density function to environmental variables, the 2p Weibull function was chosen because the estimators β and γ can be better correlated to the environmental conditions.

Furthermore, Anderson-Darling and Cramer Von Mises tests used for all ages of stands were not significant (p>0.05), indicating that the selected distribution is appropriate to describe the frequencies of the diameters. The observed values and estimates using the Weibull function are highlighted in Figure 1 and the coefficients are reported in Table 3. The properties of this function can be found in Weibull [28] and Johnson et al. [29], given by:
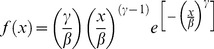
In which:

**Table 3:** Coefficients obtained for the 2p Weibull function to describe the probability of occurrence of trees as a function of diameter class in one, three, five and seven year-old stands of black wattle.

Coefficient	Year1	Year3	Year5	Year7
γ	4.115712	4.085402	3.454949	4.085136
β	5.606776	8.976419	11.455	14.10406

γ = scale parameter;

β = shape parameter;

x = diameter at breast height in cm; and

e = exponent.

Finger [4], evaluating the diameter distribution in black wattle of different ages, recommended Johnson’s SB function, while the Beta and Weibull functions yielded slightly poorer fits than those of Johnson’s SB and which can also be used for some ages. Even though Johnson’s SB function had been used successfully to describe the diametric distribution for black wattle, previous experience of the authors have not encouraged including it in this research. 2pWeibull and Gamma distributions performed the worst in the estimating the number of trees per diameter class in a study conducted by Machado et al. [30] in *Araucaria angustifolia,* results which differed from those observed for black wattle. Meanwhile Arce [31] found that the 2p Weibull distribution was flexible enough and presented excellent fit statistics for estimating diameter distributions of clones of *Populus deltoides,* further lending credibility to the observed diameter distribution in black wattle.

According to Campos and Turnbull [32], the Weibull function provides superior performance over others in many forestry applications due to its flexibility. The function has been widely applied and utilized for many forest species [25], [33], [34], [35], [36].

In fitting a probability distribution to a set of data, the hypothesis is accepted that the distribution can adequately represent a given data set [37]. This characteristic allows one to evaluate the dynamics over time through changes in the shape of the curve obtained from different coefficients according the year in question.

Over time the total amplitude of the diameters tended to increase, with the number of trees in smaller classes decreasing and increasing in the larger ones, resulting in a shift in the curve to the right. The indices of skewness and kurtosis had the effect of augmenting asymmetry with increasing age, with asymmetries becoming more negative. The observed distribution was symmetrical for the first year (year 1) and moderately asymmetrical for the other ages. Regarding the kurtosis, they can be considered leptokurtic for all ages (Table 4).

**Table 4:** Mean, minimum, maximum, median, mode, skewness and kurtosis of the diameter at breast height in one, three, five and seven year-old stands of black wattle.

Age (years)	Mean	Minimum	Maximum	Median	Mode	Asymmetry	Kurtosis
1	5.09	1.59	8.44	4.93	4.46	0.11	−0.22
3	8.15	1.27	13.05	8.91	8.28	−0.34	−0.04
5	10.29	2.55	17.19	10.90	8.28	−0.16	−0.54
7	12.79	3.66	21.49	13.05	14.32	−0.24	−0.30

**Figure 2:**
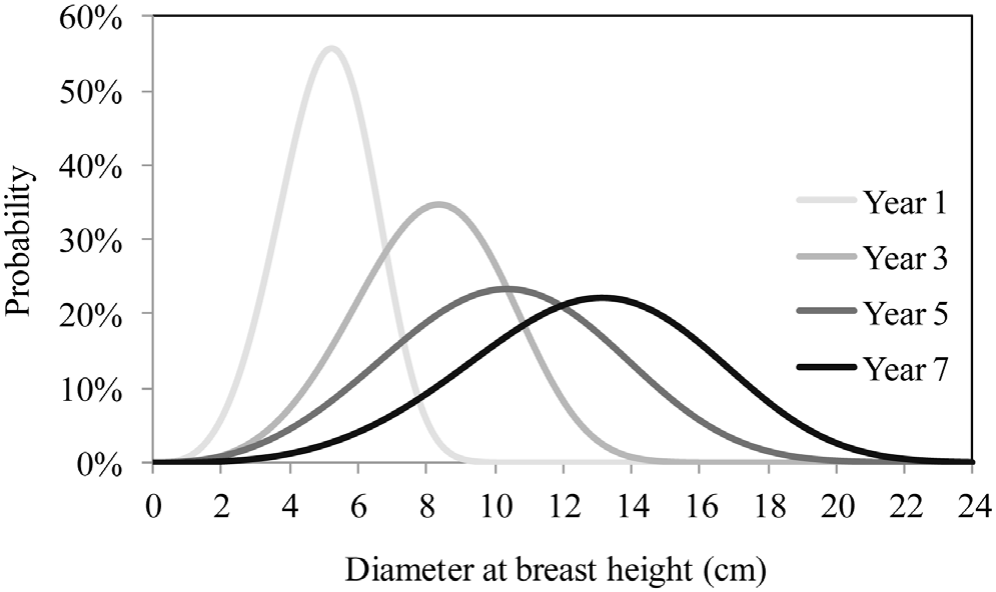
Likelihood of occurrence of trees for each diameter class as a function of the age of the black wattle stand.

To evaluate the evolution of the diameter distribution in stands of black wattle over time, and the effect of age on the curves estimated by the 2p Weibull function, curves were plotted for each age using the fitted function. At earlier stages in the stand, the frequency of individuals in smaller diameter classes is greater, but diminishes with increasing age. At the same time, as the forest grows the frequency of larger diameters increases, shifting the curve to the right (Figure 2). This is consistent with negative asymmetry.

Scolforo [38] reported a similar trend for the skewness, kurtosis and frequency of individuals as a function of diameter class in *Pinus caribaea* var. *hondurensis,* as did Arce et al. [39] in *Populus deltoides* Marsh, and Machado et al. [40] in stands of *Mimosa scabrella.* The behavior of the curves with respect to stand age is in agreement with the findings of Clutter and Bennett [41] who studied the diameter distribution in *Pinus elliottii*. The curves shifted to the right as a function of age and the number of trees in the smaller classes decreased, while in larger ones the number increased.

When the stand age curves were compared (Figure 2), significant values of Kolgomorov-Smirnov test were obtained for every stand age combination, implying that the diametric distributions change significantly after each two years of evaluation.

With the aim of evaluating the tendency between the two parameters of the Weibull function and stand age, a preliminary linear correlation analysis was carried out, yielding a negative relationship with respect to the γ estimator (r = −0.79; p≤0.018) and a positive with respect to β estimator (r = 0.97; p≤0.0005). This scenario provides evidence that the chances of successfully projecting the probability distribution are improved through recovery of function coefficients, as per Nogueira et al. [42], Leite et al. [43], Binotti et al. [44], Retslaff et al. [45] and specifically in stands of black wattle per Maestri [5]. Even though only few points were used to establish the correlations, the authors mentioned above had successfully achieved better results increasing the number of pairs for such estimators.

An important research focus in this work is the correlation between the parameters of the distribution function with environment variables. Stronger correlations improve the chances of success in the use of the distribution in simulations, starting from the premise that the involvement of environmental variables increases the capacity of the probabilistic function for generalization. The effects of the parameters γ and β on the distribution are very different, since γ affects the symmetry, while β affects the kurtosis. Machado et al. [40] report that measures of skewness and kurtosis describe the shape and evolution of the distribution curves, in which the asymmetry is the degree of deviation from the normal curve and kurtosis is the degree of flatness or height of a given distribution relative to the Normal distribution.

Through a simple linear regression analysis correlations between the two parameters of the Weibull function and meteorological and soil surface horizon elements can be established. Regarding the parameter γ the highest correlations were observed for accumulated rainfall, global solar radiation, relative humidity, hydrogen + aluminum CEC, CEC pH and ISMP. As for the β estimator the highest correlations were observed for precipitation and global solar radiation (Table 5).

**Table 5:** Correlation between the parameters of the 2p Weibull function to describe the diameter frequency in one, three, five and seven years old stands of black wattle with meteorological and soil surface chemical elements.

Variable	Correlation with γ	Variable	Correlation with β
Rainfall	−0.78	pH	−0.44
Solar radiation	−0.73	ISMP	−0.34
Air moisture	−0.65	CEC Base	−0.28
Hydrogen+aluminum	−0.62	Magnesium	−0.22
Aluminum	−0.61	Calcium	−0.08
CEC pH	−0.61	Organic matter	0.05
Clay	−0.48	Potassium	0.07
CEC Al	−0.42	Average Temperature	0.18
Organic matter	−0.40	Phosphorus	0.21
Minimum Air Temperature	−0.08	Maximum Temperature	0.21
Calcium	0.02	Clay	0.22
Phosphorus	0.03	CEC pH	0.27
Potassium	0.06	Minimum Temperature	0.31
Average Air Temperature	0.07	H+Al	0.32
Maximum Air Temperature	0.08	Air Moisture	0.40
Magnesium	0.20	Al	0.41
CEC Base	0.30	CEC+Al	0.42
pH	0.64	Solar Radiation	0.97
ISMP	0.68	Rainfall	0.98

The relationship between the estimators of the Weibull function (2 parameters) and soil chemical and meteorological elements was also analyzed by stepwise variable selection technique. Draper and Smith [23] report that Stepwise Multiple Regression is one of the most recommended for effectively selecting explanatory variables. Using the *stepwise* method to obtain the coefficient β resulted in three steps, where the first variable to enter the model was the accumulated rainfall (Prec), the second was air temperature (Tavg), and finally the SMP index (ISMP).

In the first step the equation β = 4.29692+0.00106 Prec was obtained, and the fit yielded a coefficients of determination of 95.89%. In the second step the equation obtained was β = −13.41109+0.00106 Prec+0.95961 Tmed, resulting in a coefficients of determination of 98.63%. Finally the equation β = −15.70246–1.04422 ISMP+0.001 Prec+1.41365 Tmed was obtained, with a coefficient of determination of 99.66%.

In the second step the average temperature was revealed to be not significant at 1% probability, as was the case of ISMP in the third step, and should thus be excluded from the model. Given the very small improvement in the performance of the equation afforded by the addition of the variables Tavg and ISMP, we elected the model resulting from step 1, which includes only the variable most highly correlated with the parameter β, i.e. rainfall. As for this equation all coefficients were significant (p<0.01) and met all the conditions of regression tests that were subject to the Durbin-Watson (2.03), White (3.24) and Shapiro-Wilk (0.98) tests.

For the coefficient γ the *stepwise* method resulted in two steps, the first including rainfall (Prec) and the second cumulative solar radiation (Rg). In step 1 the equation obtained was γ = 5.62235–0.00020493 Prec, which resulted in a coefficient of determination of 60.91%. In step 2, the equation obtained was γ = 5.01823–0.00194 Prec+0.00043431 Rg, with a coefficient of determination of 89.67%.

Despite improvement of over 28% in the coefficient of determination with the inclusion of the variable Rg, the model obtained in step 2 presented problems of multicollinearity given that variance inflation factor was greater than 10. Multicollinearity occurs when any independent variable is highly correlated with another independent variable as in this case.

Given this situation, the equation obtained in the first step was selected, which met all the condition tests of the regression, given the significance of all coefficients (p<0.01) and not significant (p>0.05) according to the Durbin-Watson (2.36), White (0.13) and Shapiro-Wilk (0.92) tests. Although only eight points composed the data set used in this study, this finds suggest that the equation is suitable to estimate the Weibull parameters, since all the equation’s coefficients were significant and the equation met all the requirements of regression analysis. Increasing the sample could improve the goodness of fit and make the equation even more reliable.

Therefore, the models obtained both for β and for parameter γ were equal, i.e. included only the cumulative rainfall. In these equations, the estimators of the Weibull function and rainfall data were related to the planting site (municipalities of Piratini and Cristal) and were subjected to regression analysis with a *dummy* variable. This suggests that a single equation could be fit, and planting site need not be considered since the *dummy* used to evaluate the effect of planting site was not significant (p>0.05).

**Figure 3:**
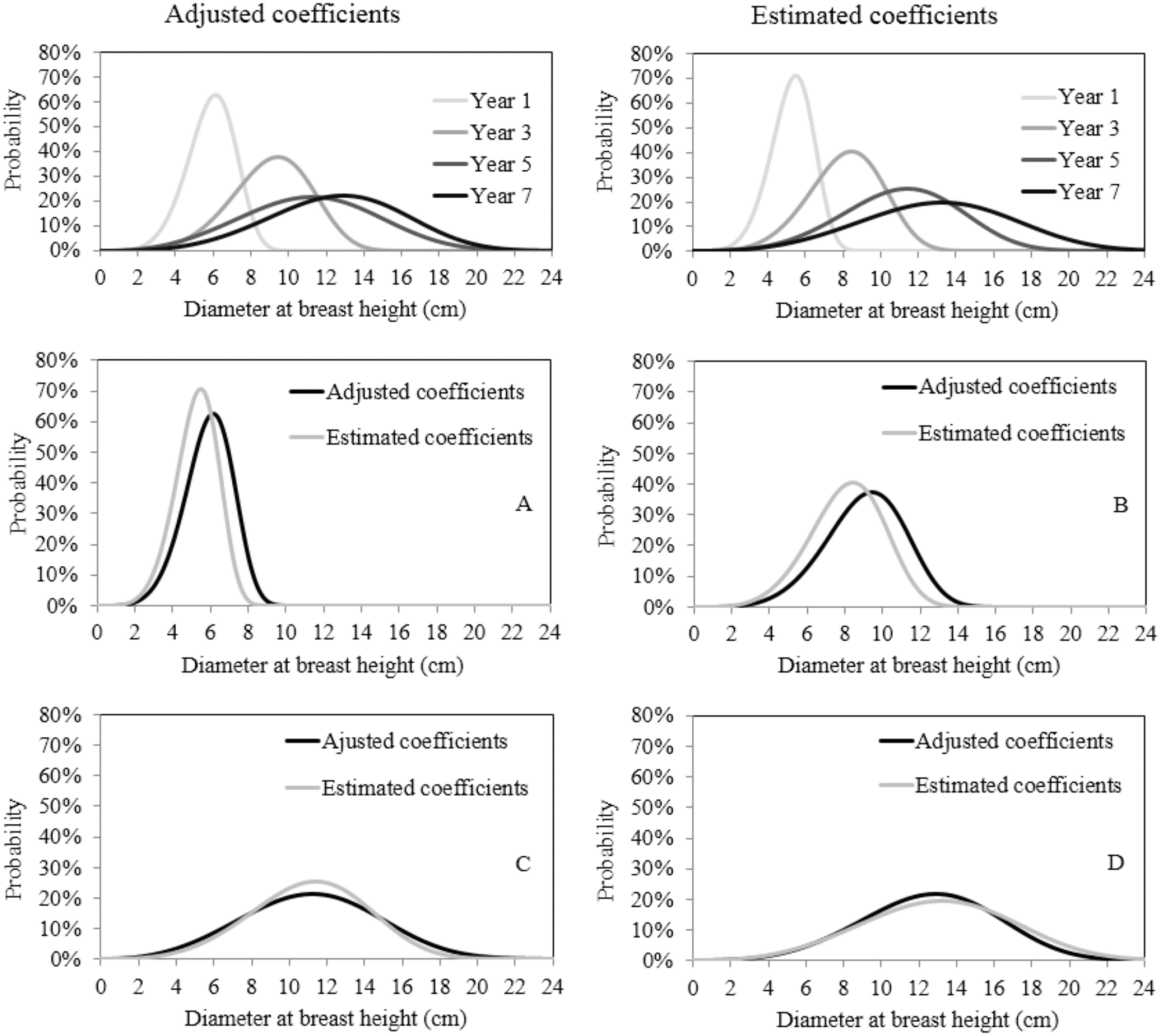
Diameter distribution obtained by directly fitting the 2p Weibull function (Year i) and diameter distribution obtained via the prediction of parameters of the 2p Weibull function in year (A), three (B), five (C) and seven (D) years old stands of black wattle located in the municipality of Cristal.

**Figure 4:**
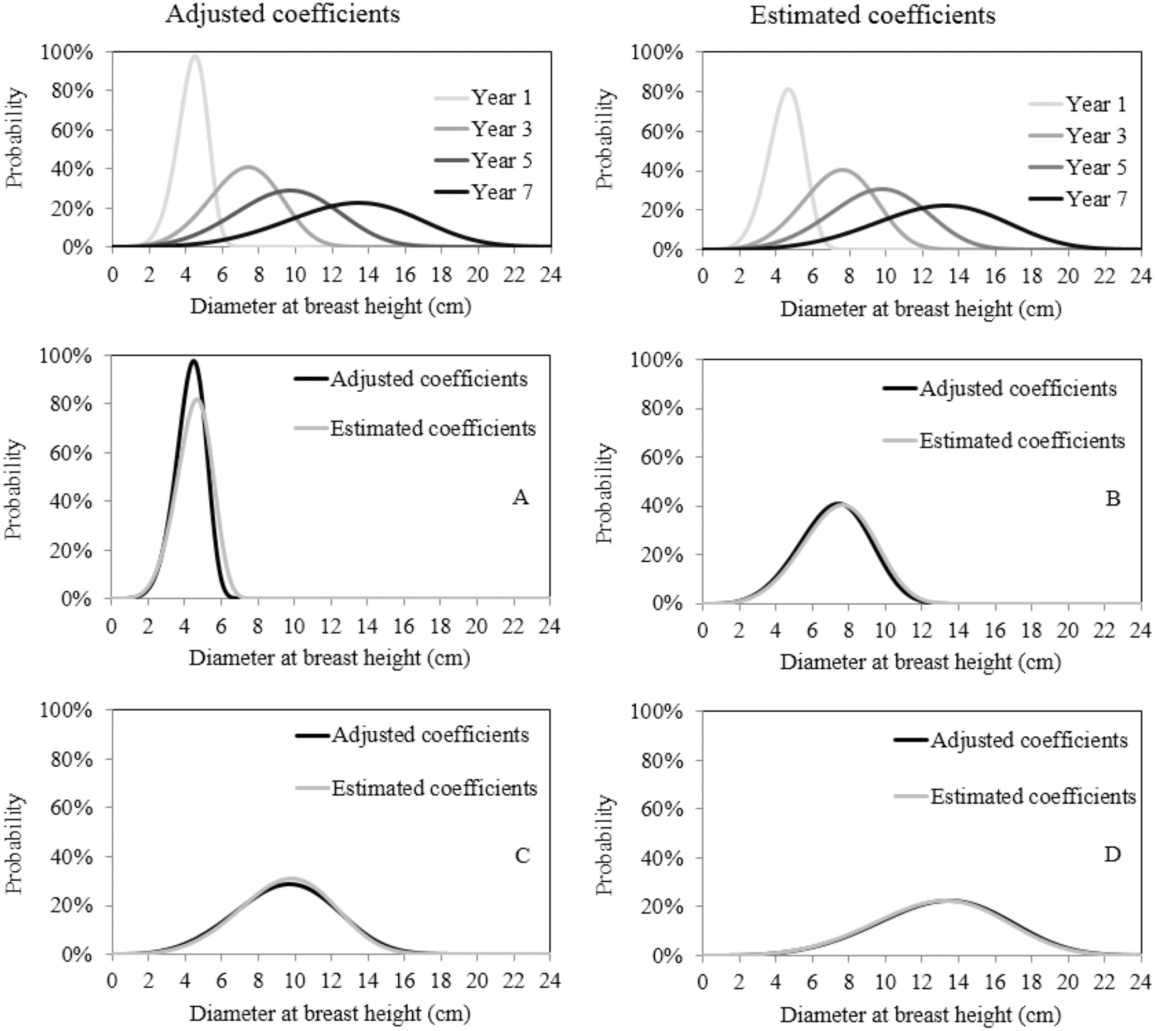
Diameter distribution obtained by directly fitting the 2p Weibull function (Year i) and diameter distribution obtained via the prediction of parameters of the 2p Weibull function in year (A), three (B), five (C) and seven (D) years old stands of black wattle located in the municipality of Piratini.

To evaluate the performance of the resulting equations, the curves obtained by estimating the parameters of the Weibull distribution using the equations generated by the *stepwise* procedure were compared with those obtained via the direct fit of the function by the *capability* procedure. The Kolgomorov-Smirnov test was used for this purpose, though values of KS were not significant (p>0.01), implying the absence of significant differences between the two curves (Figures 3 and 4). Thus, the estimation of the parameters of the Weibull function using accumulated rainfall proved adequate despite the small data set used in this study.

This relationship is consistent because the growth in diameter, and consequently the diameter distribution, depends on the amount of water available during the growth cycle and agrees with the findings of research conducted by several authors. Allen and Albaugh [46] highlighted that in *Pinus taeda* the low water availability and extreme temperatures negatively affect the leaf area and hence reduce interception and use of solar radiation. Thus, the growth tends to be slower, since it depends on the amount of photosynthetically active radiation intercepted.

Other authors [47], [48], [49] emphasize that water is one of the factors that most limits forest productivity by controlling the opening and closing of stomata, the absorption of nutrients from the soil and the medium in which the chemical and biochemical reactions of photosynthesis occurs. According to Binkley et al. [50] a leaf with an adequate water supply is able to fix more carbon per unit of light intercepted than a leaf suffering from water stress and closed stomata. Other studies, similar to those conducted with eucalyptus, report elevated net primary productivity with increased water availability in the forest, resulting in greater light interception and photosynthetic efficiency [50], [51], [52].

Stape [53] concluded that rainfall is highly related to eucalyptus yields, explaining 80% of variation of the mean annual increment in diameter. Other studies, such as Benson et al. [54], Snowdon and Benson [55], Samuelson et al. [56] and Williams and Gresham [57], in trials with irrigation, also observed the dependence of the diameter growth with water availability in the soil.

Thus, the close connection between the parameters of the 2pm Weibull distribution and rainfall seems coherent. As it was seen, the growth of a forest stand is dependent on the availability of water, represented by the equation:
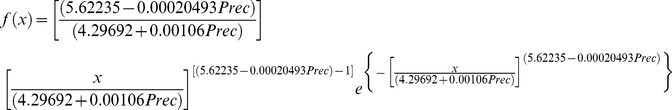



In which:

Prec = accumulated rainfall in mm;

x = diameter at breast height in cm; and

e = exponent.

Having obtained this model in which the Weilbull function estimators are dependent on rainfall, important applications are implied, such as simulating the growth potential in areas without records, as well as the behavior of a planting when faced with different environmental conditions. It may also be used to project diameter, starting from the basis of the rainfall during a given interval of time affecting the forest.

**Figure 5:**
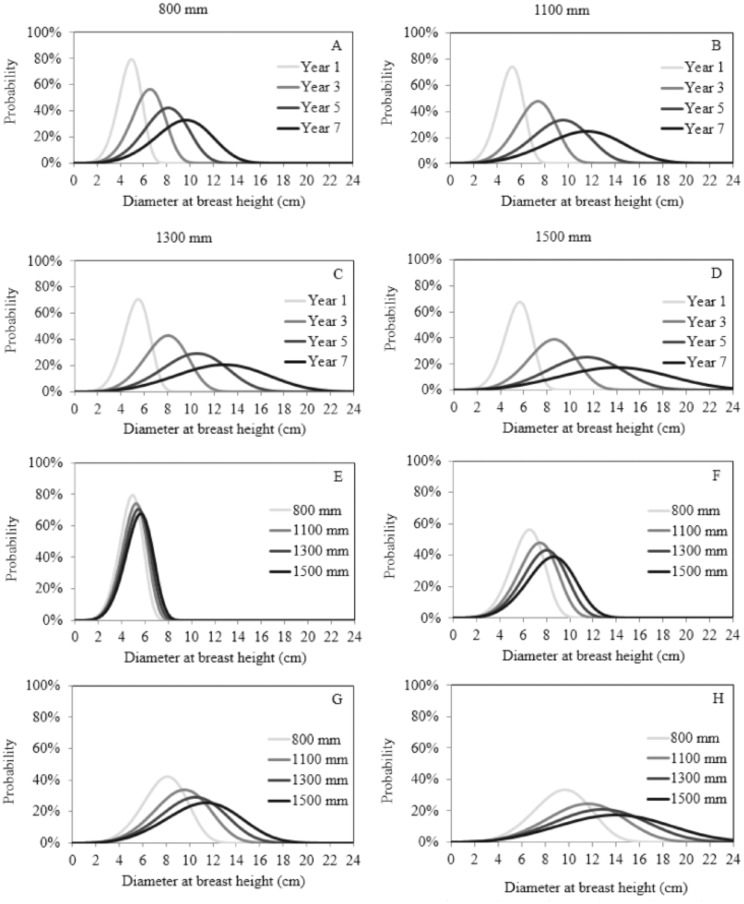
Simulation of diameter distribution in black wattle stands under different rainfall scenarios (A–D). Where: E = one year-old stand, F = three year-old stand, G = five year-old stand, and H = seven year-old stand.

A simulation was performed in order to explore different rainfall regimes: 800, 1100, 1300 and 1500 mm of rainfall per year. Results indicate that stands subjected to reduced rainfall regimes tend to have higher frequencies of trees with smaller diameter, while stands under high rainfall regimes tend to have higher frequencies of trees with large diameters (Figure 5).

## Conclusions

The Gamma, Weibull 2p and Weibull 3p functions are flexible enough to represent the distributions of diameters of black wattle over all ages of the stands.

The 2p Weibull function was selected to describe the probability of occurrence of individuals as a function of diameter class, which revealed that:

The total amplitude of diameters increases as the age of the stand increases. Also the number of trees in smaller classes decreases while the number larger classes increases, causing the curve to shift to the right.
Indices of skewness and kurtosis indicate an increase in the asymmetry with increasing age, such that asymmetries become more negative.
The distribution was symmetrical for the first year (year 1), moderately asymmetrical for the other ages, and the curves are leptokurtic.
The curves show statistically different distributions between the ages of the stands.
The two parameters of the Weibull function are correlated with the age of the stand, as well as accumulated rainfall.
Rainfall influences the diameter distribution, and therefore the relationships obtained from the Weibull function estimators can be used to perform simulations. A priori, it is not necessary to consider the planting site in such a relationship, since no differences between the planting sites were identified.
Using simulation, it was possible to detect that stands subject to less intense rainfall regimes tend to have higher probabilities of frequencies of trees with smaller diameter, while stands subject to higher rainfall regimes tend to have higher frequencies of trees with larger diameters.

Having obtained this model in which the Weilbull function estimators are dependent on rainfall, important applications are implied, such as simulating the growth potential in areas without records, as well as the behavior of a planting against different environmental conditions. It may also be used to make diameter prognosis, considering the rainfall effect on the forest during a given interval of time.

Although few data had been used to obtain the correlations and to fit the regression models, the results were enough to indicate the relationships between the environmental variables and the 2p Weibull coefficients. However, this fact presents a certain limitation regarding the consistency of statistical estimators. In future works such relationships can be improved by increasing the sampling intensity.
